# A Nationwide Survey of Training Pathways and Practice Trends of Endoscopic Submucosal Dissection in Canada

**DOI:** 10.1093/jcag/gwac037

**Published:** 2023-01-24

**Authors:** Suqing Li, Jeffrey Mosko, Gary May, Christopher Teshima

**Affiliations:** Division of Gastroenterology and Hepatology, Department of Medicine, University of Calgary, Calgary, Alberta, Canada; Division of Gastroenterology and Hepatology, Department of Medicine, The Center for Advanced Therapeutic Endoscopy and Endoscopic Oncology, St. Michael’s Hospital, Toronto, Ontario, Canada; Division of Gastroenterology and Hepatology, Department of Medicine, The Center for Advanced Therapeutic Endoscopy and Endoscopic Oncology, St. Michael’s Hospital, Toronto, Ontario, Canada; Division of Gastroenterology and Hepatology, Department of Medicine, The Center for Advanced Therapeutic Endoscopy and Endoscopic Oncology, St. Michael’s Hospital, Toronto, Ontario, Canada; Division of Gastroenterology and Hepatology, Department of Medicine, The Center for Advanced Therapeutic Endoscopy and Endoscopic Oncology, St. Michael’s Hospital, Toronto, Ontario, Canada

**Keywords:** Endoscopic practice, Endoscopic submucosal dissection, Luminal endoscopy

## Abstract

**Background:**

Endoscopic submucosal dissection (ESD) has become an established standard for endoscopic removal of large gastrointestinal (GI) lesions and early GI malignancies. However, ESD is technically challenging and requires significant health care infrastructure. As such, its adoption in Canada has been relatively slow. The practice of ESD across Canada remains unclear. Our study aimed to provide a descriptive overview of training pathways and practice trends of ESD in Canada.

**Methods:**

Current ESD practitioners across Canada were identified and invited to participate in an anonymous cross-sectional survey.

**Results:**

Twenty-seven ESD practitioners were identified; survey response rate was 74%. Respondents were from 15 different institutions. All practitioners underwent international ESD training of some type. Fifty per cent pursued long-term ESD training programs. Ninety-five per cent attended short-term training courses. Sixty per cent and 40% performed hands-on live human upper and lower GI ESD, respectively, before independent practice. In practice, 70% saw an increase per year in number of procedures performed from 2015 to 2019. Sixty per cent were dissatisfied with their institution’s health care infrastructure to support ESD. Thirty-five per cent perceived their institution as supportive of expanding the practice of ESD.

**Conclusions:**

Several challenges exist to the adoption of ESD in Canada. Training pathways are variable, with no set standards. In practice, practitioners express dissatisfaction with access to necessary infrastructure and feel poorly supported in expanding the practice of ESD. As ESD is increasingly the accepted standard for the treatment of many neoplastic GI lesions, greater collaboration between practitioners and institutions is crucial to standardize training and ensure patient access.

## BACKGROUND

Endoscopic submucosal dissection (ESD), first pioneered in Japan, has allowed for curative endoscopic *en bloc* resection of large superficial gastrointestinal (GI) lesions and early GI malignancies. ESD provides clear advantages for removing large or advanced GI lesions compared to other methods, with lower recurrence rates compared to endoscopic mucosal resection and lower cost, morbidity, and mortality compared to surgery (1,2). Despite this, the adoption of ESD has been limited in North America owing to various factors, including steep learning curves, limited expertise, and inadequate remuneration (3).

The indications for ESD are broadening to include dysplastic Barrett’s esophagus, superficial intramucosal esophageal adenocarcinoma, squamous cell carcinoma, early gastric cancer, and large colorectal neoplasms limited to the mucosa or superficial submucosa (3). ESD is increasingly regarded as a potential first-line option for the treatment of early malignant and large benign gastrointestinal lesions in place of surgery, with curative resection rates up to 89% (4,5). Although highly effective in expert hands, ESD is associated with significant risks of perforation and bleeding dependent on the lesion, patient, and endoscopist factors (2,6). Given differences between patient volumes, endoscopist training, and health care infrastructure available for the practice of ESD between Asian and Western countries, it is unclear whether previously reported outcomes for ESD from studies primarily conducted in Japan are consistent with its use in Canada.

In Japan, unlike in North America, there are many centers with sufficient expertise and volume to take trainees (7). Given these limitations, there remains no standardized pathway to gaining competency in North America. As such, only a handful of centers and endoscopists across Canada have enough experience to practice ESD. A prior study surveying endoscopists performing ESD in Canada gave a preliminary overview of barriers to uptake. However, their findings were limited by the survey distribution at the early stages of ESD adoption in Canada and the small number of active ESD practitioners, with only 11 respondents actively performing ESD (8). There has since been a significant increase in the number of ESD practitioners in Canada. With the growing recognition and acceptance of ESD, it is essential to evaluate its use in Canada thoroughly, particularly given our country’s unique multicultural population, differences in training opportunities, and health care model (6). Our study provides an updated comprehensive survey of endoscopists actively practicing ESD across Canada as of 2021 and an in-depth review of training pathways, growth trends, and practice patterns.

## METHODS

### Study Design

A cross-sectional survey of ESD training pathways, practice environment and trends was created and reviewed by the study team. The study team consisted of three therapeutic endoscopists (J.M., G.M., C.T.) actively performing ESD at the largest referral center for therapeutic endoscopy in Canada, where up to 100 ESD procedures are performed per year. The survey was divided into four general categories, evaluating (i) training history, (ii) ESD experience and practice environment, (iii) ESD technique/equipment, and (iv) health care infrastructure for ESD (Supplementary Appendix 1). The survey was created on the SurveyMonkey platform for distribution.

An up-to-date contact list of all endoscopists currently taking referrals for ESD at centers across Canada was generated by contacting known practitioners from internal networks and all tertiary Canadian gastroenterology departments for ESD practitioners at their respective sites. Identified endoscopists were contacted via e-mail for recruitment and provided a link to the survey on SurveyMonkey. Participants were made clear that the survey was anonymous and de-identified. Survey data collection was conducted from October 2020 to May 2021. Remuneration was not provided for survey completion. This study was approved by the Unity Health REB.

### Analysis

Raw data were extracted from SurveyMonkey. Descriptive statistics and qualitative analysis were conducted via Microsoft Excel and SPSS v28.0 (IBM Corp).

### Data Availability

The data underlying this article will be shared on reasonable request to the corresponding author.

## RESULTS

The survey was distributed to 27 endoscopists identified as actively practicing ESD in Canada. One endoscopist indicated they were no longer performing ESD. Twenty endoscopists actively performing ESD completed the survey with a survey completion rate of 74% (*n* = 20/27).

### Participant Demographics

Median age of respondents was 43 years (IQR 8.5). Ninety-five per cent (*n* = 19/20) of respondents were male. Ten respondents practiced in Ontario, five practiced in Quebec, one practiced in Manitoba, one practiced in Saskatchewan, one practiced in Alberta, and one practiced in British Columbia.

Median number of years in independent practice at the time of survey was 10.5 years (IQR 8.8). Median number of years of independent ESD practice at the time of survey was 3.5 years (IQR 2.8).

### ESD Training History

Ninety per cent (*n* = 18/20) of respondents completed a traditional therapeutic endoscopy fellowship program (Table 1). During therapeutic endoscopy fellowship 94% (*n* = 17/18) received training in ERCP and/or EUS. Twenty-two per cent (*n* = 4/18) received training in ESD during therapeutic endoscopy fellowship. Fifty per cent (*n* = 10/20) underwent a formal ESD training program outside Canada. Median duration of training programs was 2 months (IQR 5.8). Ninety-five per cent of respondents (*n* = 19/20) attended short-term ESD training courses. Median number of courses attended was 2 (IQR 1.3). All respondents received international ESD training outside of Canada in some form. Ninety-five per cent (*n* = 19/20) received ESD training on animal models. Sixty-five per cent (*n* = 13/20) performed ESD on live human cases before independent practice; of which 54% (n = 7/13) received exposure on both upper and lower GI ESD, 38% (*n* = 5/13) on upper GI ESD only, and 8% (*n* = 1/13) on lower GI ESD only. As the primary operator in live human cases prior to independent ESD practice, 15% (*n* = 2/13) of respondents performed >20, 8% (*n* = 1/13) performed 11 to 20, 77% (*n* = 10/13) performed 1 to 10 cases.

**Table 1.** Respondents training history

Survey *f*ield	Participant responses
Underwent traditional advanced therapeutic endoscopy fellowship	90% (*n* = 18/20)
Trained in ERCP + EUS	61% (*n* = 11/18)
Trained in ERCP Only	11% (*n* = 2/18)
Trained in EUS Only	22% (*n* = 4/18)
Received ESD *t*raining during advanced ­fellowship	22% (*n* = 4/18)
Attended short-term ESD training courses/workshops	95% (*n* = 19/20)
Median number of attended courses/workshops	2 (IQR 1.3)
Attended courses/workshops outside of ­Canada	100% (18/18)
Received ESD training on animal models	95% (*n* = 19/20)
Underwent long-term* ESD training program	50% (*n* = 10/20)
Median duration of training program (Months)	2 (IQR 5.8)
Received hands-on experience during ­program (versus observation only)	80% (*n* = 8/10)
Performed ESD on human cases prior to ­independent practice	65% (*n* = 13/20)
Both upper and lower GI ESD	35% (*n* = 7/20)
Upper GI ESD *o*nly	25% (*n* = 5/20)
Lower GI ESD *o*nly	5% (*n* = 1/20)

^*^Defined as >30 days

In general, 50% (*n* = 10/20) received ESD training via a combination of short-term courses and animal model training and 45% (*n* = 9/20) via short-term courses and a long-term formal ESD training program. One participant received training solely via a long-term formal ESD training program that lasted 14 months.

**Figure 1.**
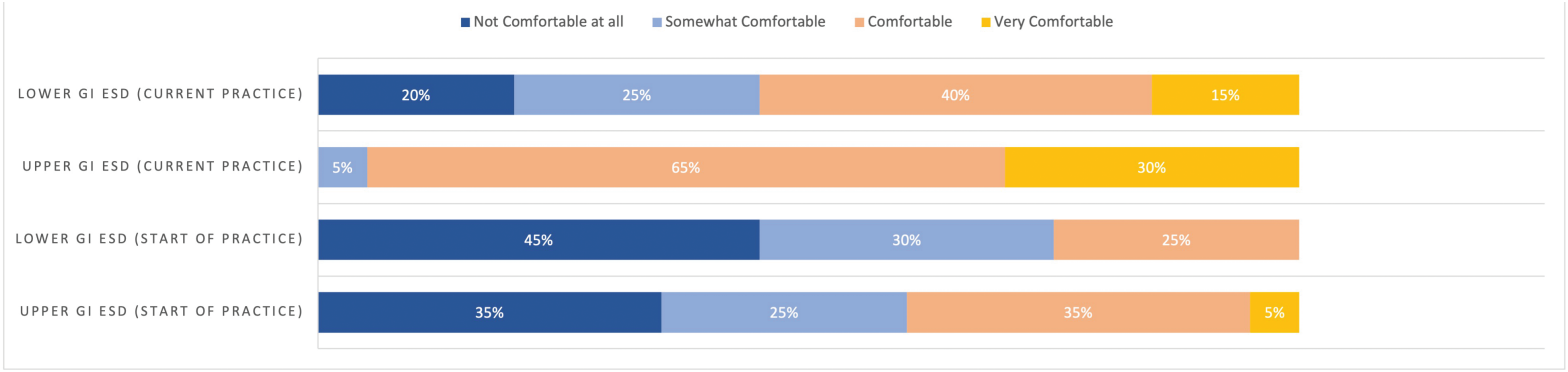
Respondents comfort level with performing ESD.

At the start of practice, 35% (*n* = 7/20) and 45% (*n* = 9/20) of respondents reported feeling ‘not comfortable at all’ with performing upper GI and lower GI ESD, respectively, independently. Only 35% (*n* = 7/20) and 25% (*n* = 5/20) reported feeling ‘comfortable’ with performing upper GI and lower GI ESD, respectively, independently at the start of practice. However, comfort level at the time of the survey was reported as ‘comfortable’ or ‘very comfortable’ by 95% (*n* = 19/20) of practitioners for upper GI ESD and 55% (*n* = 11/20) for lower GI ESD (Figure 1).

### Independent ESD Practice Environment

**Figure 2.**
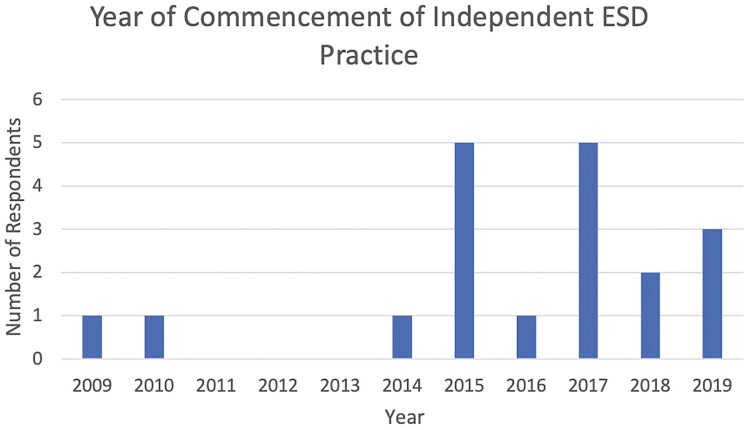
Year of commencement of ESD practice.

**Figure 3.**
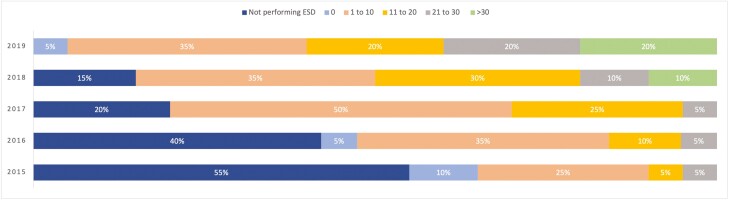
Approximate number of ESD procedures performed by respondents per year.

Eighty-five per cent (*n* = 17/20) of respondents began independent ESD practice after 2014 (Figure 2). The median number of practitioners offering ESD at each center was 1. As shown in Figure 3, the approximate number of ESD procedures performed by respondents each year starting from 2015 has sequentially increased, with only two respondents performing >10 cases per year in 2015 and twelve respondents performing >10 cases per year by 2019.

Fifty-eight per cent (*n* = 11/19) of respondents reported an approximate wait time for ESD of 1 to 3 months. The median duration of time usually booked for lower and upper GI ESD cases were 120 minutes (IQR 60) by respondents. Sixty per cent (*n* = 12/20) of respondents performed ESD in the endoscopy suite, and 35% (*n* = 7/20) performed ESD in an operating room. Seventy-five per cent (*n* = 15/20) of respondents performed ESD under monitored anesthesia care with propofol or general anesthesia. See Table 2 for further details.

**Table 2.** ESD practice environment

Survey field	Participant responses
Median number of ESD practitioners at center	1 (range 1*–*3)
Approximate wait-time for ESD	
<2 weeks	11% (*n* = 2/19)
2*–*4 weeks	26% (*n* = 5/19)
1*–*3 months	58% (*n* = 11/19)
3*–*6 months	5% (*n* = 1/19)
Median duration of time usually booked for ESD cases (minutes)	
Upper GI	120 (IQR 60)
Lower GI	120 (IQR 60)
Usual location for performing ESD	
Endoscopy Suite	60% (*n* = 12/20)
Operating Room	35% (*n* = 7/20)
Most common sedation used for ESD	
MAC with Propofol	15% (*n* = 3/20)
General *a*nesthesia	60% (*n* = 12/20)
Conscious *s*edation (opioids/benzos)	20% (*n* = 4/20)

**Figure 4.**
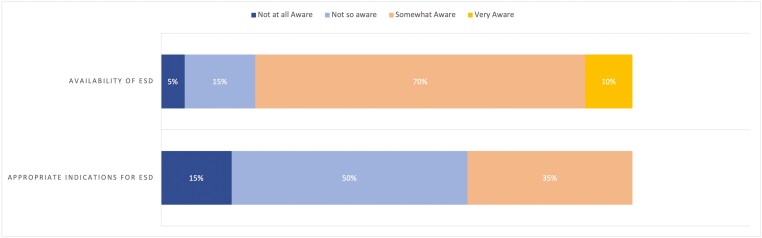
Referral providers awareness of ESD availability and indications.

The most common sources of ESD referrals were from surgeons (95% [*n* = 19/20]) or gastroenterologists (90% [*n* = 18/20]). Only 10% (*n* = 2/20) of respondents reported referral providers were ‘very aware’ of the availability of ESD in their area and 65% (*n* = 13/20) were ‘not so aware’ or ‘not at all aware’ of the appropriate indications for ESD referral (Figure 4).

### Health Care Infrastructure for ESD

**Figure 5.**
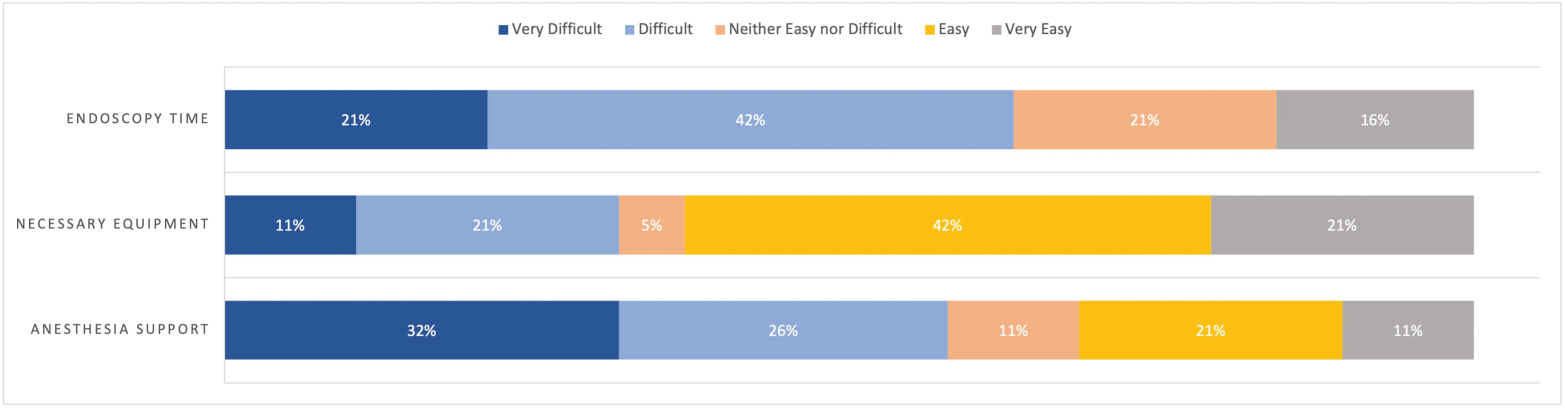
Ease of securing resources for ESD.

**Figure 6.**
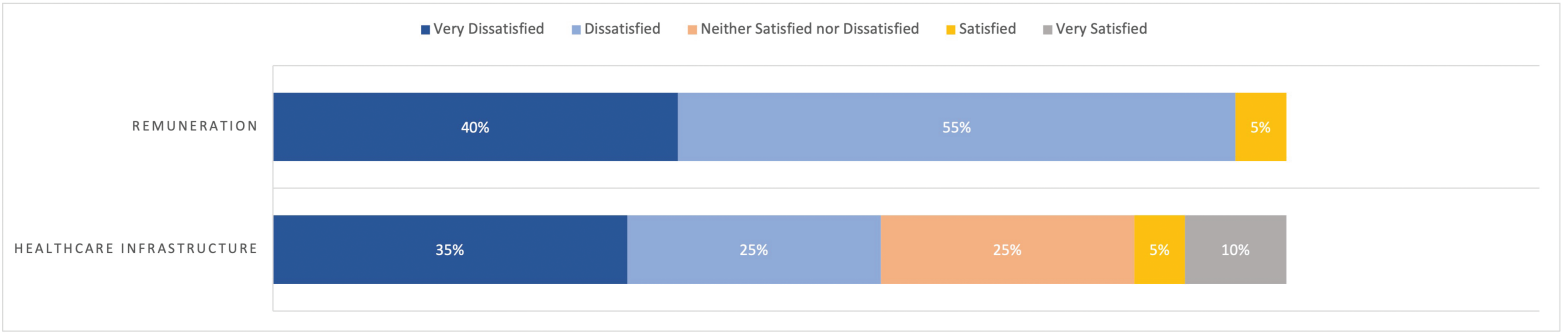
Overall satisfaction with remuneration and infrastructure for ESD.

**Figure 7.**
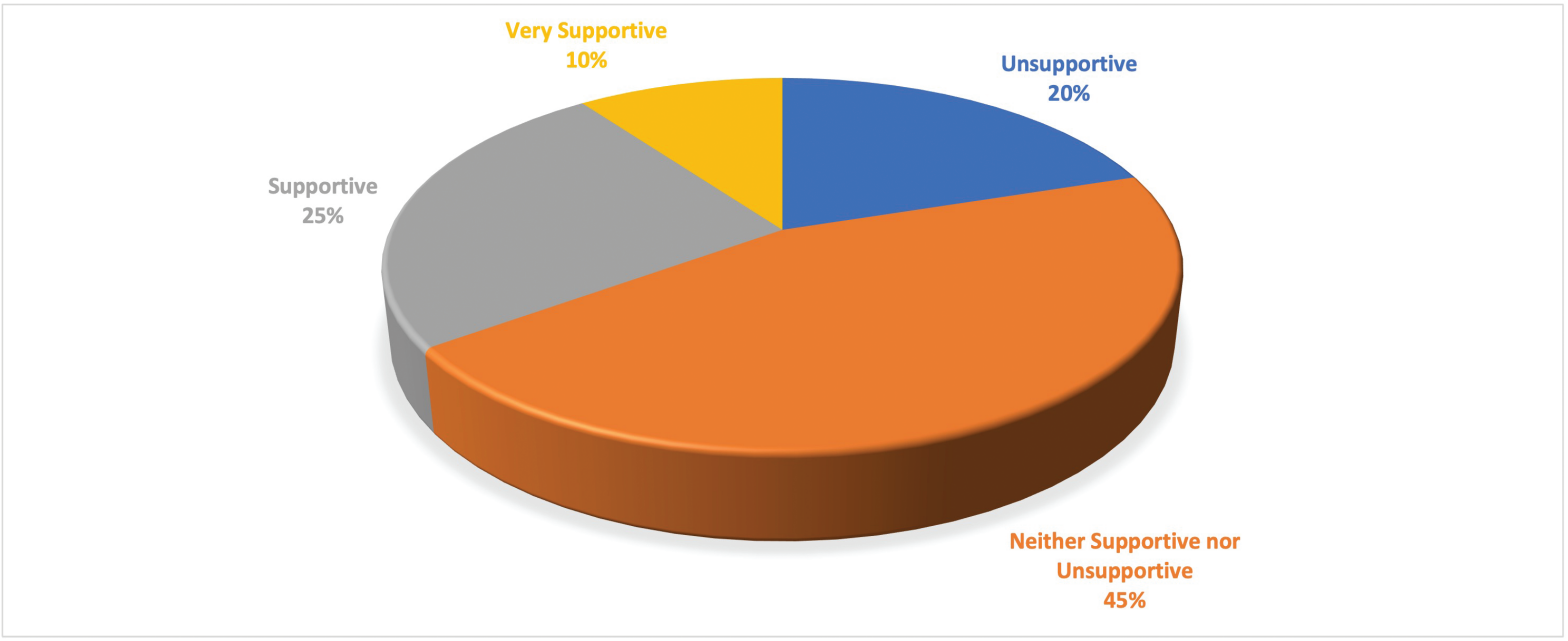
Perceived supportiveness of institution in expanding practice of ESD.

Most respondents reported difficulty in securing endoscopy time and anesthesia support for ESD (Figure 5). However, most respondents reported relative ease in securing necessary technical equipment (Figure 5). Ninety-five percent (*n* = 19/20) of respondents were either dissatisfied or very dissatisfied with the remuneration for ESD, and the majority (55% [*n* = 11/20]) were overall not satisfied with their center’s current health care infrastructure to support their ESD practice (Figure 6). Notably, only 35% (*n* = 7/20) felt their institution was either ‘supportive’ or ‘very supportive’ in expanding the practice of ESD (Figure 7).

## DISCUSSION

The results of our study have provided an up-to-date overview of the evolution of the state of ESD practice in Canada. Our results demonstrate significant heterogeneity in ESD training pathways with no standard routes that are mainstream currently. We have also shown considerable growth in the number of ESD practitioners and the number of ESD cases being performed over the past few years. Despite this growth, however, there appears to be relative dissatisfaction among practitioners regarding the overall health infrastructure, remuneration and institutional support for ESD practice in Canada.

ESD training pathways remain variable, as evidenced by our results and remain a significant barrier to adopting ESD in North America, as supported by prior studies (9,10). Multiple reasons have been raised that precluded the development of standard training pathways in Western countries, including the availability of training/supervision, obtaining adequate case-loads, and obtaining the necessary equipment and infrastructure (10). As such, many endoscopists have traditionally looked toward visiting Japanese centers for training and/or attending international courses by expert ESD practitioners (10,11). In addition, our findings demonstrated that many practitioners felt uncomfortable performing ESD at the start of independent ESD practice, which may reflect the lack of structured training curriculums and guidance. However, as more western endoscopists have become proficient in ESD, there is now an opportunity to develop standardized curriculums in Western centers. To this end, the American Society of Gastrointestinal Endoscopy and the European Society of Gastrointestinal Endoscopy has recently published guidance statements for developing core curriculums for ESD training (9,12). In Canada, the availability of ESD training remains limited, with only six respondents currently providing hands-on ESD training based on our survey results. However, as ESD continues to grow, we expect the barriers to accessing ESD training in Canada will significantly decrease.

As speculated by Rai et al., there has been considerable growth in the number of ESD operators since their survey, with 20 respondents actively performing ESD responding to our study versus 11 respondents back in 2018 (8). Additionally, our survey results show that respondents progressively had increasing numbers of ESD procedures performed yearly. This likely reflects increasing recognition and referrals for ESD; however, as evidenced by our results, there remains relatively poor awareness of appropriate indications for ESD from referral providers. This is understandable given the complexities surrounding the selection of the best resection technique for the appropriate lesion (13). The American Gastroenterological Association and the Japanese Gastroenterological Endoscopy Society have previously published guidance regarding appropriate indications (3,14,15). However, with the nuances of lesion selection, some groups have proposed the development of regional multi-disciplinary teams (MDT) for decisions regarding lesion management (10). The development of MDTs may promote the growth of appropriate ESD referrals and increase patient access to this specialized service (16).

Lastly, our survey results show a perceived lack of institutional support and health care infrastructure for the practice and growth of ESD in Canada. This may have in part stemmed from earlier skepticism regarding the efficacy and safety of ESD in Western regions, particularly given most outcome studies were based in Japan (13,17). However, Draganov et al. recently published the largest prospective multicenter study on the outcomes of ESD in North American centers (including one Canadian center) of over 600 patients. Their results demonstrated the safety and efficacy of ESD in North America, with outcomes adhering to established consensus quality standards (17). The need for resources such as specialized equipment, endoscopy time and anesthesia support has also been a known barrier to the growth of ESD (18). This challenge in accessing resources remains evident in our survey results. However, several studies have now demonstrated the cost savings of ESD when performed on appropriate lesions as it allows the avoidance of surgical intervention (19–21). In public health care systems such as Canada, the proper implementation of ESD provides an opportunity to provide cost-effective care for the benefit of both patients and taxpayers and thus should be regarded as a priority for governments and health care systems.

## CONCLUSION

There remain several challenges in adopting ESD in Canada, ranging from the lack of structured training pathways and perceived deficiencies in institutional support and health care infrastructure. However, there has been evident growth in the practice of ESD in Canada. This will undoubtedly continue to expand as ESD has become the standard of care for the resection of many neoplastic GI lesions. To ensure adequate patient access to care and the maintenance of high-quality practice standards, increased partnership with institutions and health care networks are crucial to facilitate the successful practice of ESD in Canada.

## Supplementary Material

gwac037_suppl_Supplementary_Appdendix
