# Supplementary material for: A Nationwide Survey of Training Pathways and Practice Trends of Endoscopic Submucosal Dissection in Canada
Source: J Can Assoc Gastroenterol. 2023 Jan 24;6(2):80–5. doi: 10.1093/jcag/gwac037 (PMC10071294; doi:10.1093/jcag/gwac037)
Supplement: gwac037_suppl_Supplementary_Appdendix [file gwac037_suppl_supplementary_appdendix.docx]

**CANADIAN ESD SURVEY**

| **Participant Demographics** | |
| --- | --- |
| 1. Age (Years) |  |
| 1. Gender | M  F |
| 1. Number of years in practice |  |
| 1. City and Province of current practice in Canada |  |

| **Endoscopy and Training History** | |
| --- | --- |
| 1. Year of **core** gastroenterology residency completion |  |
| 1. No. of years of **core** gastroenterology residency |  |
| 1. City and Country of your **core** gastroenterology residency |  |
| 1. Completion of advanced therapeutic fellowship training after core training | Y  N |
| If Yes,   - 1. No of years of advanced therapeutic fellowship |  |
| - 1. Country of advanced therapeutic fellowship? |  |
| - 1. Were you trained in ERCP during your fellowship? | Y  N |
| - 1. Were you trained in EUS during your fellowship? | Y  N |
| - 1. Did you receive any training in ESD during your fellowship? | Y  N |
| If Yes, Type of Training (check all that apply): | |
| - Observation Only |  |
| - Hands-on Animal Model |  |
| - Hands-on Live Human Cases |  |
| - If hands-on experience, approximate number of live human ESD procedures performed under supervision during this program? | |
| *Upper GI* | 0  1-10  11-20.  >20 |
| *Colorectal* | 0  1-10  11-20  >20 |
| - If hands-on experience, approximate number of animal model ESD procedures performed under supervision during this program? | |
| *Upper GI* | 0  1-10  11-20  >20 |
| *Colorectal* | 0  1-10  11-20  >20 |
| 1. Completion of ESD training courses/workshop offered outside of a traditional fellowship | Y  N |
| if YES,  no. of workshops/courses completed |  |
| Country/Countries of ESD training courses/workshop attended or completed | Country 1:  Country 2:  Country 3:  Country 4:  Country 5: |
| 1. ESD training on animal models received | Y  N |
| If YES, Approximate No. of ESDs performed on an animal model |  |
| Live Pig Model |  |
| *Upper GI* | 0  1-10  11-20  >20 |
| *Colorectal* | 0  1-10  11-20  >20 |
| Ex Vivo Model |  |
| *Upper GI* | 0  1-10  11-20  >20 |
| *Colorectal* | 0  1-10  11-20  >20 |
| 1. Did you undergo international post-fellowship training in ESD? | Y  N |
| If Yes: | |
| - 1. Country of post-fellowship ESD training |  |
| - 1. Year of commencement of this training |  |
| - 1. Duration of this extra training (months) |  |
| Type of international post- fellowship training: | |
| - Observation only |  |
| - Observation and Hands-on experience |  |
| - If hands-on training, approximate number of ESD cases performed under supervision during this program? | |
| *Upper GI* | 0  1-10  11-20  >20 |
| *Colorectal* | 0  1-10  11-20  >20 |
| 1. Total no. of human ESDs you were in **primarily observation/assistant** prior to practicing ESD independently? |  |
| - 1. *Upper GI* | 0  1-10  11-20  >20 |
| - 1. *Colorectal* | 0  1-10  11-20  >20 |
| 1. Total no. of **supervised human ESDs** performed (**primary operator for majority of procedure**) prior to practicing ESD independently |  |
| - 1. *Upper GI* | 0  1-10  11-20  >20 |
| - 1. *Colorectal* | 0  1-10  11-20  >20 |
| 1. What was your comfort level with ESD at **the start of independent practice**? 2. Upper GI ESD: 3. Colorectal ESD: | Very Comfortable  Comfortable  Somewhat Comfortable  Not Comfortable At All |
| 1. What is your current comfort level with ESD? 2. Upper GI ESD: 3. Colorectal ESD: | Very Comfortable  Comfortable  Somewhat Comfortable  Not Comfortable At All |

| **ESD Experience and Practice Environment** | |
| --- | --- |
| 1. What year did you start practicing ESD independently? |  |
| 1. What year did your center start offering ESD? |  |
| 1. How many ESD practitioners are at your center? |  |
| 1. In total approximately how many ESD procedures did you perform in: |  |
| Calendar year 2015 | 0  1-10  11-20  21-30  31-50  >50 |
| Calendar year 2016 | 0  1-10  11-20  21-30  31-50  >50 |
| Calendar year 2017 | 0  1-10  11-20  21-30  31-50  >50 |
| Calendar year 2018 | 0  1-10  11-20  21-30  31-50  >50 |
| Calendar year 2019 | 0  1-10  11-20  21-30  31-50  >50 |
| 1. How many ESD cases for **each indication** did you perform in **calendar year 2018?** | |
| Barrett’s dysplasia & esophageal intramucosal adenocarcinoma | 0  1-5  6-10  11-20  >20 |
| Esophageal squamous dysplasia & SCC | 0  1-5  6-10  11-20  >20 |
| Advanced gastric dysplasia & early gastric cancer | 0  1-5  6-10  11-20  >20 |
| Duodenal lesions | 0  1-5  6-10  11-20  >20 |
| Colorectal polyps & superficial cancer | 0  1-5  6-10  11-20  >20 |
| 1. How many ESD cases for **each indication** did you perform in **calendar year 2019?** | |
| Barrett’s dysplasia & Esophageal intramucosal adenocarcinoma | 0  1-5  6-10  11-20  >20 |
| Esophageal squamous dysplasia & SCC | 0  1-5  6-10  11-20  >20 |
| Advanced gastric dysplasia & early gastric cancer | 0  1-5  6-10  11-20  >20 |
| Duodenal lesions | 0  1-5  6-10  11-20  >20 |
| Colorectal polyps & superficial cancer | 0  1-5  6-10  11-20  >20 |
| 1. Which physicians send you referrals for patients receiving ESD (Check all that apply)? | Primary care providers (GP/GIM)  Surgeon  Medical specialist/Radiologist/Oncologist  Gastroenterologist  Self- referral |
| 1. What is the awareness of the **availability of ESD** by referral providers in your region? | Very Aware  Aware  Somewhat Aware  Not Aware |
| 1. What is the awareness of **appropriate indications for ESD** by referral providers in your region? | Very Aware  Aware  Somewhat Aware  Not Aware |

| **Technical Features** | |
| --- | --- |
| 1. What is your most commonly used electrosurgical knives for ESD (Check all that apply)? | Dual Knife (Olympus)  IT Knife (Olympus)  IT2 Knife (Olympus)  IT Nano Knife (Olympus)  TT Knife (Olympus)  Hook Knife (Olympus)  Flush Knife (Fujinon)  Mucosectom (Pentax)  B-Knife (Zeon Medical)  HybridKnife I-Type (ERBE)  HybridKnife T-Type (ERBE)  Other |
| 1. What is your most commonly used injection solution for ESD | Hyaluronic Acid with Methylene Blue/Indigo Carmine  Normal Saline with Methylene Blue/Indigo Carmine  Eleview  Orise Gel  LiftUp  Voluven with Methylene Blue  Other |
| 1. Do you add epinephrine to injection solution? | No  Yes Always  Sometimes |
| 1. What is the usual Time Duration you book for Upper GI ESD Cases? |  |
| 1. What is the usual Time Duration you book for Colorectal GI ESD Cases? |  |
| 1. Where do you usually perform upper GI ESD? | OR  Endoscopy Suite |
| 1. Where do you usually perform lower GI ESD? | OR  Endoscopy Suite |
| 1. What sedation do you most commonly use to perform ESD? | General Anesthesia  MAC with Propofol  Conscious sedation with Opioids/Benzodiazepines  other (please specify) |
| 1. How satisfied are you with the **availability of necessary technical equipment** to perform successful ESD at your center? | Very Satisfied  Satisfied  Neither satisfied nor dissatisfied  Dissatisfied  Very dissatisfied |

| **Healthcare Infrastructure** | |
| --- | --- |
| 1. How easy is it to secure endoscopy time in your unit for ESD? | Very Easy  Easy  Neither easy nor difficult  Difficult  Very difficult |
| 1. How easy is it to secure the necessary equipment for ESD at your center? | Very Easy  Easy  Neither easy nor difficult  Difficult  Very difficult |
| 1. How easy is it to secure anesthesia support for ESD at your center? | Very Easy  Easy  Neither easy nor difficult  Difficult  Very difficult |
| 1. What is the current wait-time for ESD in your practice? | <2 weeks  2-4 weeks  1-3 months  3-6 months  >6 months |
| 1. Do you have billing codes for the ESD procedures that you perform? | Yes No  Other: |
| 1. How satisfied are you with renumeration for ESD? | Very Satisfied  Satisfied  Neither satisfied nor dissatisfied  Dissatisfied  Very dissatisfied |
| 1. Overall how satisfied are you with the current healthcare infrastructure to support ESD in your practice? | Very Satisfied  Satisfied  Neither satisfied nor dissatisfied  Dissatisfied  Very dissatisfied |
| 1. How supportive is your institution for expanding the practice of ESD? | Very supportive  Supportive  Neither supportive nor unsupportive  Unsupportive  Very Unsupportive |
| 1. Does your institution currently maintain a prospective database of ESD cases and outcomes? | Yes  No |
| 1. Are you providing any hands-on ESD training to your current gastroenterology residents or fellows? | Yes  No |

| **Survey Completion** |
| --- |
| Thank you for completing our survey, to track completion and your consent, please click the following link below to enter your name and e-mail to ensure your responses remain de-identified from your survey responses: ___________ (Link to exit survey) |
